# Biofiltration of toluene in the presence of ethyl acetate or *n*-hexane: Performance and microbial community

**DOI:** 10.1371/journal.pone.0302487

**Published:** 2024-05-07

**Authors:** Xiaojuan Xue, Hai Wang, Jian Zhai, Xujun Nan

**Affiliations:** 1 School of Environmental Engineering, Gansu Forestry Polytechnic, Tianshui, Gansu province, People’s Republic of China; 2 Department of printing and packaging Engineering, Shanghai Publishing and Printing College, Shanghai, People’s Republic of China; Shahrood University of Technology, ISLAMIC REPUBLIC OF IRAN

## Abstract

This study describes the operation of two independent parallel laboratory-scale biotrickling filters (BTFs) to degrade different types of binary volatile organic compound (VOC) mixtures. Comparison experiments were conducted to evaluate the effects of two typical VOCs, i.e., ethyl acetate (a hydrophilic VOC) and *n*-hexane (a hydrophobic VOC) on the removal performance of toluene (a moderately hydrophobic VOC) in BTFs ‘‘A” and ‘‘B”, respectively. Experiments were carried out by stabilizing the toluene concentration at 1.64 g m^−3^ and varying the concentrations of gas-phase ethyl acetate (0.85–2.8 g m^−3^) and *n*-hexane (0.85–2.8 g m^−3^) at an empty bed residence time (EBRT) of 30 s. In the presence of ethyl acetate (850 ± 55 mg m^-3^), toluene exhibited the highest removal efficiency (95.4 ± 2.2%) in BTF “A”. However, the removal rate of toluene varied from 48.1 ± 6.9% to 70.1 ± 6.8% when 850 ± 123 mg m^-3^ to 2800 ± 136 mg m^-3^ of *n*-hexane was introduced into BTF “B”. The high-throughput sequencing data revealed that the genera *Pseudomonas* and *Comamonadaceae_unclassified* are the core microorganisms responsible for the degradation of toluene. The intensity of the inhibitory or synergistic effects on toluene removal was influenced by the type and concentration of the introduced VOC, as well as the number and activity of the genera *Pseudomonas* and *Comamonadaceae_unclassified*. It provides insights into the interaction between binary VOCs during biofiltration from a microscopic perspective.

## Introduction

In recent years, the concentration of fine particles (PM_2.5_) has decreased gradually in China. However, it still exceeds the World Health Organization’s annual mean concentration limit of 5 μg m^-3^ [1]. Additionally, ozone (O_3_) pollution is becoming increasingly prevalent [2]. Volatile organic compounds (VOCs) are widely recognized as major precursors of O_3_ and PM_2.5_, which have a serious impact on environmental quality and human health [3]. Toluene (T), ethyl acetate (EA), and *n*-hexane (H) are among the most frequently used as industrial solvents, and are major pollutants found in the exhaust air of printing facilities [4, 5]. Various methods have been employed to reduce the amount of VOCs in the air, including recovery technologies such as absorption, condensation, adsorption, and membrane separation, as well as destruction technologies like incineration, photocatalytic, catalytic combustion, plasma catalysis, and biological degradation [6]. Metabolic activities of microorganisms can transform VOCs into carbon dioxide (CO_2_), H_2_O, and biomass, providing a low energy demand and environmentally friendly solution for the abatement of VOCs in polluted air emissions with high flow rates and low pollutant contaminants [7]. Such biological techniques including biofiltration, biotrickling filtration and bioscrubbing have been extensively utilized. The bio-trickling filter (BTF) is equipped with a continuous spraying system to trickle and recycle the minimal salt medium (MSM) over the packing bed. It can supply the necessary nutrients, and control pH and moisture. Additionally, it can timely and efficiently drain away toxic metabolites and prevent their accumulation, thereby facilitating the degradation process. In recent years, BTF has become the leading technique due to its advantages of being capable of treating recalcitrant VOCs and acidic or alkaline compounds, generating no secondary pollution, and having low operating and capital costs and low pressure drop during long-term operation [8, 9]. The elimination of VOCs by BTF is influenced by several factors, including the concentration and physical properties of the VOCs, their biodegradability, the empty bed residence time (EBRT), the specific surface area of the packed medium, nutrition rate, and liquid trickling rate [10].

However, applying BTFs to deal with VOCs from industries presents challenges due to the variability of flow rate and composition of contaminants. Industrial emissions typically consist of mixtures of VOCs with varying rates of biodegradation. For instance, in printing companies, the composition of exhaust gas may vary depending on the specific processes involved. In industrial applications, BTFs may encounter changes in major substrates, such as a shift from a T and EA mixture to a T and H mixture, or vice versa. Moreover, the treatment of multiple pollutants with BTFs encountered the challenge of substrate interactions, which can be antagonistic, neutral, or synergistic [11]. A number of research contributions have examined the simultaneous abatement of a binary mixture of hydrophobic and hydrophilic VOCs [12, 13]. These studies have shown that the efficiency of BTF in eliminating hydrophobic VOCs is enhanced by the existence of hydrophilic VOCs. In the biofiltration of a binary mixture of VOCs, hydrophilic VOCs are degraded preferentially. However, few studies systematically compare the effects of introducing various water-soluble VOCs on T biofiltration.

Therefore, the objective of this study was to evaluate the removal performance of BTFs in removing various binary mixtures and to lay the foundations for future industrial applications. First, T was selected as a model compound to represent moderately hydrophobic compounds. EA and H, as different water-soluble VOCs, were introduced into two parallel BTFs mixed with T, respectively, to gain insights into the interactions between multiple VOCs during biodegradation. This may provide a theoretical basis for the industrial application of BTFs. In addition, the structure and diversity of the bacterial communities in the BTFs were analyzed using high-throughput sequencing technology. This will provide a microbial ecology perspective on the performance differences of BTFs when introducing VOCs with different Henry’s constants.

## Materials and methods

### VOCs and MSM

The model VOC pollutants used in the biodegradation experiments were T, EA, and H (analytical reagent, >99.5%, Nanjing Chemical Reagent Co. Ltd., China). Their Henry’s constants and other related physical properties are listed in Table 1 [14]. The components of the MSM were previously described by Li et al. [15].

**Table 1:** Henry’s constants and other related physical properties for the selected VOCs.

VOC	Boiling Point (°C)	Vapor Pressure (mm Hg at 20°C)	Solubility (g/L at 20°C)	Dimensionless Henry’s Constant (25°C)
**T**	110.8	22	0.52	0.29
**EA**	77	73	80	5.48 × 10^−3^
**H**	68.7	120	0.013	53

### BTF set up

**Fig 1:**
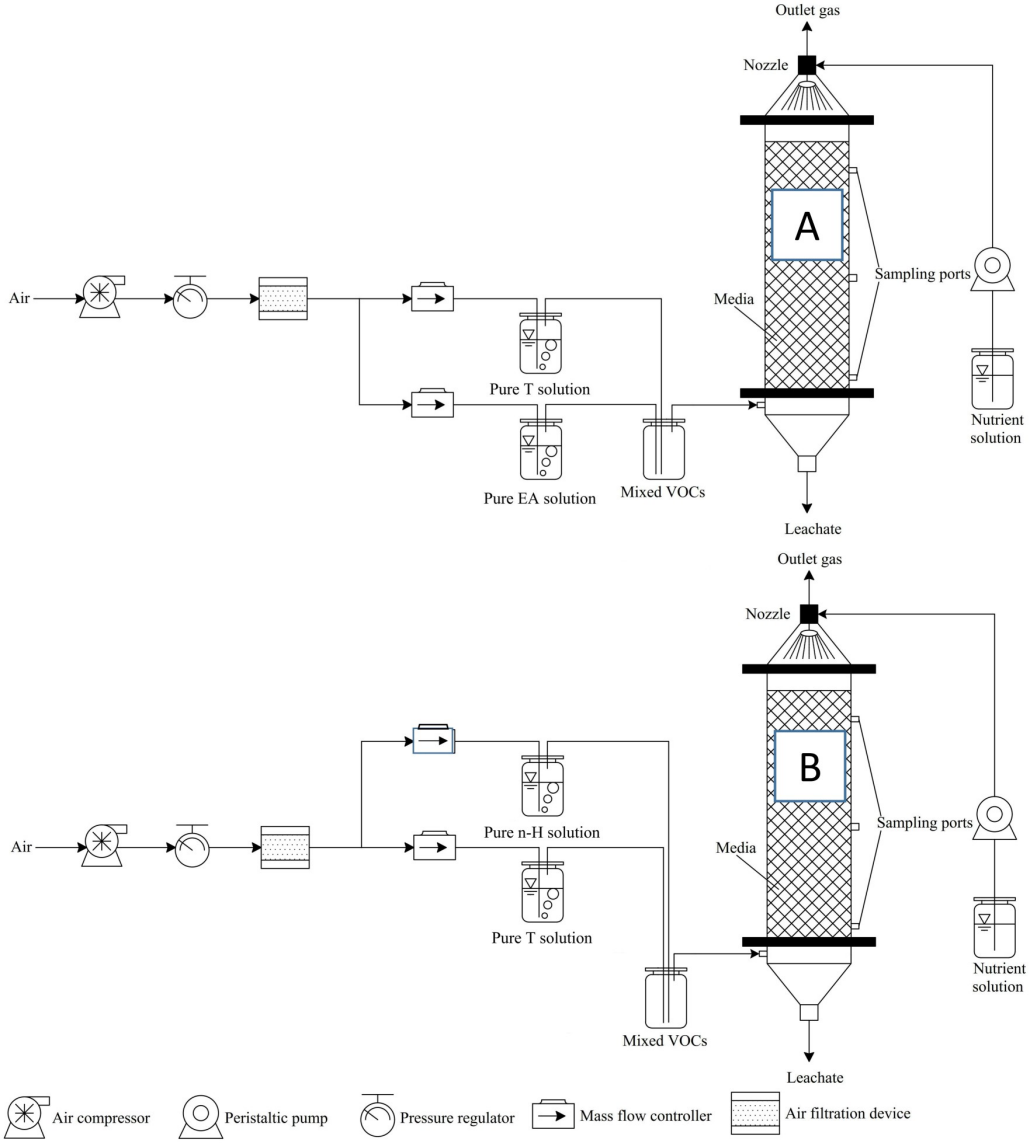
Schematic representation of the experimental setup.

A schematic diagram of the experimental set-up is shown in Fig 1. An air pump introduced the compressed air stream into the system, which was then divided into two paths precisely controlled by two air mass flow meters (CS200, Beijing Sevenstar Flow Co., LTD., China). One air flow passed through the vessel containing T, while the other bubbled through liquid EA. After mixing in a tank, these two streams were fed into the BTF “A” in an up-flow mode. Following the same procedure, another binary mixture of T and H was created to be introduced into the BTF “B”.

The BTF was constructed using translucent Plexiglas with an internal diameter of 7 cm, a bed height of 60 cm and an effective volume of 2.3 L. Polyurethane foam (1 cm^3^) was used as the packing material, which was randomly packed. To achieve uniform spraying of MSM, a nozzle was installed at the top of the column. The packing was supported by a perforated plate at the bottom, which can also be used to ensure even distribution of the gas mixture. Gas sampling ports with rubber septa are located at heights of 0 cm, 20 cm, 40 cm, and 60 cm along the column from bottom to top. These ports are used to collect packing samples using tweezers for bioinformation analysis and to collect gas samples by gas-tight syringes (250 μL, Hamilton, Switzerland) to determine the concentrations of T, EA, H, and CO_2_ at different heights of the outlet. Each sample was analyzed in triplicate.

### BTF inoculation and operation

The BTFs were inoculated with activated sludge from the secondary sedimentation tank of the Sinopec Yangzi Petrochemical Company wastewater treatment plant (WWTP) located in Nanjing, China. The mixed liquor volatile suspended solids (MLVSS) value of the activated sludge was 2000 mg L^-1^. The mixed culture was recirculated through the packing layers using a peristaltic pump (WT600-1F, Baoding Longer Pump Co., Ltd., China) at a flow rate of 0.5 L min^-1^ until visible biomass appeared on the surface of the carrier material. Throughout the experiment, EBRT and the operating temperature were maintained at 30 s and room temperature (20–25°C), respectively. The trickling density (F), which is defined as the volume of MSM flowing through a unit cross-sectional area of the bio-trickling filter per unit time, was kept at 0.81 m^3^ m^-2^ h^-1^. It can be expressed as follows:

$$Trickling\;density\;(m^{3}\;m^{-2}\;h^{-1}):F=4Q_{l}/\pi D^{2}$$ (1)

where F is the trickling density (m^3^ m^-2^ h^-1^). Q_l_ is the spray liquid flow rate (m^3^ h^-1^), D is the diameter of the filter bed (m).

The original pH of MSM was adjusted to 7.0. The moisture content of the packing layers was maintained at a level of 75–85% through continuous spraying of MSM. To control the excessive biomass accumulation in gas-phase BTFs, weekly backwashing was performed with 1 L of 20°C water from the top of the BTFs for 1 hour [16].

After inoculation and acclimation, the BTF “A” was initially fed with different proportions of T_EA gaseous mixtures. During phase I, the BTF “A” was operated only with T (1640 mg m^-3^). During phase II, phase III, and phase IV, various concentrations of EA (850 mg m^-3^, 1750 mg m^-3^ and 2800 mg m^-3^) were fed to the BTF “A” together. The strategy of operation for BTF “B” exactly followed the sequence applied for BTF “A”, except for changing the target VOCs to T_H. The experimental conditions are summarized in Table 2.

**Table 2:** BTFs operating experimental conditions.

BTF “A” Phase	Time (day)	T concentrations (mg m^-3^)	EA concentrations (mg m^-3^)	BTF “B” Phase	Time (day)	T concentrations (mg m^-3^)	H concentrations (mg m^-3^)
**I**	16–60	1640 ± 40	-	**I**	16–60	1640 ± 37	-
**II**	61–105	1640 ± 40	850 ± 55	**II**	61–105	1640 ± 136	850 ± 123
**III**	106–150	1640 ± 10	1750 ± 80	**III**	106–150	1640 ± 34	1750 ± 22
**IV**	151–195	1640 ± 18	2800 ± 100	**IV**	151–195	1640 ± 270	2800 ± 136

### Analytical methods

Gas samples were analyzed for T, EA and H concentrations using a gas chromatograph (GC) (2014, SHIMADZU, Japan) equipped with a Rtx-5 column (30 m, 0.32 mm ID, 0.5 μm) and a flame ionization detector (FID). The flow rates for H_2_ and air were 30 mL min^−1^ and 300 mL min^−1^, respectively, with nitrogen used as the carrier gas at a flow rate of 20 mL min^−1^. The temperatures at the GC injection, oven, and detection ports were maintained at temperatures of 150°C, 120°C, and 250°C, respectively. The analysis of CO_2_ was performed using a GC equipped with a methane conversion furnace, a packed column, and a FID (9890A, Shanghai Linghua Instrument Co., Ltd., China). Total organic carbon (TOC) was measured with a TOC analyzer (TOC 5000A, Shimadzu, Japan). The pH value of MSM was measured with a pH meter (FEP20, Mettler-Toledo, Switzerland). Total nitrogen (TN) was determined using a TNM-1 total nitrogen meter (Shimadzu, Kyoto, Japan).

The performances of the BTF were estimated by the following equations:

$$Empty\;bed\;residence\;time\;(s):EBRT=V/Q_{g}\times 3600$$ (2)

$$Removal\;efficiency\;(\%):RE=(C_{o}-C_{i})/C_{o}\times 100\%$$ (3)

$$Inlet\;loading\;rate\;(g\;m^{-3}\;h^{-1}):ILR=Q_{g}C_{o}/V$$ (4)

$$Elimination\;capacity\;(g\;m^{-3}\;h^{-1}):\;EC=Q_{g}(C_{o}-C_{i})\;/\;V$$ (5)

where *C*_*o*_ and *C*_*i*_ are the inlet and outlet concentrations (mg m^-3^) of T, EA and H, respectively. *Q*_*g*_ is the gaseous mixture flow rate (m^3^ h^-1^), *V* is the volume of the filter bed (m^3^).

### Mineralization and carbon balance analysis

The mixed VOCs are generally aerobically degraded into CO_2_, H_2_O, intermediates and biomass by microorganisms during the biodegradation process. After 10 days of operation for each phase, T-RE of the BTFs tended to stabilize. Subsequently, a carbon balance analysis was conducted.

$$\begin{array}{c} Mineralization\;rate\;(\%):MR=(C_{CO_{2},out}-C_{CO_{2},in})\\ \times M_{VOC}/(n\times C_{VOC,in}\times M_{CO_{2}})\times 100\% \end{array}$$ (6)

$$m_{o}=m_{i}+m_{CO_{2}}+m_{biomass}+m_{intermediates}$$ (7)

where C_CO2,out_, C_CO2,in_, n and C_VOC,in_ are the outlet and inlet CO_2_ concentrations (mg m^-3^), the carbon number of VOC, and the inlet VOC concentration (mg m^-3^), respectively. M_VOC_ and M_CO2_ represent the molar mass of VOC and CO_2_, respectively. The m_o_ and m_i_ represent the carbon contents of the influent and effluent mixed VOCs, respectively. The m_CO2_ is the carbon content of the outlet CO_2_, with its background value deducted. The m_biomass_ refers to the carbon content of the proliferative biomass on the packings, while the m_intermediates_ represents the carbon content of the intermediates dissolving in the leachate within a cycle of 48 hours. The m_o_, m_i_ and m_CO2_ can be calculated based on the influent and effluent concentrations of two binary gas mixtures, as well as the CO_2_ outlet concentration. The dissolved CO_2_ in the leachate was disregarded due to its lower solubility in water compared to T and acetone vapors. The wet weight of biomass in the BTFs was periodically determined using a weighing method described in the literature [17]. An electronic scale was utilized for weighing. The m_biomass_ was calculated by subtracting the weight on the sampling day from the initial weight (day 0). The m_intermediates_ can be obtained from the TOC of the leachate. The carbon recovery, R, is defined as the percentage ratio of the sum of m_i_, m_CO2_, m_biomass_ and m_intermediates_ to m_o_.

### Microbial analysis

#### Sample collection and DNA extraction

For microbial community analysis, some polyurethane foam cubes were collected from the middle layer of the BTF at designed intervals. Each sample was immersed in 20 mL of sterilized water and sonicated for 20 s using an ultrasonic oscillator (DL-1800D, Shanghai Wuxiang Instrument Co., Ltd., China) to strip the biomass from the packing material. The samples were then centrifuged at 15,000 rpm for 10 min. The DNA was extracted using a FastDNA^®^ Spin Kit for Soil (MP Biomedicals, CA, America) following the manufacturer’s instructions.

#### Polymerase Chain Reaction (PCR)

PCRs were conducted in 20 μL reaction mixtures containing 10 ng of template DNA, 4 μL of 5×FastPfu Buffer, 2 μL of 2.5 m mol L^−1^ dNTPs, 0.8 μL of each primer (5 μ mol L^−1^), 0.4 μL of FastPfu Polymerase and complemental ddH_2_O. Primer set 338F / 806R (5’-ACTCCTACGGGAGGCAGCAG-3’ / 5’-GGACTACHVG-GGTWTCTA AT-3’) was chosen for PCR of 16S rRNA. The DNA was amplified by using a GeneAmp^®^ (9700, ABI, America). PCR amplification was performed by initially denaturing for 3 min at 95°C, followed by cycles of denaturation for 30 s at 95°C, annealing for 30 s at 55°C, extension for 45 s at 72°C and a final extension for 10 min at 72°C. The PCR of 16S rRNA required 27 cycles. The amplicons were extracted from 2% agarose gels and purified using an AxyPrep DNA Gel Extraction Kit (Axygen Biosciences, Union City, CA, America) according to the manufacturer’s instructions and quantified using QuantiFluor^™^-ST (Promega, America).

#### Illumina high-throughput sequencing and bioinformatics analysis

The purified amplicons were pooled in equimolar contents and sequenced in paired-end mode (2×250) on an Illumina MiSeq PE250 platform (Shanghai Majorbio Bio-pharm Technology Co.,Ltd., China). Using Mothur (http://www.mothur.org/), corresponding sequences with specific barcodes within each sample were selected. Data denoising was then conducted following the method described in a previous study [18]. Finally, these samples were compared at the same sequencing depth. The Ribosomal Database Project (RDP) (http://rdp.cme.msu.edu/) classifier was used for downstream taxonomic assignment with a confidence threshold of 50%. Mothur was applied to calculate the richness and diversity indices, including the operational taxonomic units (OTUs), Chaos index, and Shannon index [19]. Sequences that were more than 97% similar were classified into a single operational taxonomic unit based on a distance matrix. A cluster analysis of the microbial community was conducted by using Paleontological Statistics (PAST, v.3.01) software with an unweighted pair-group average method.

### Functional analysis

To comprehend the functional changes of microorganisms in BTFs, first, the Phylogenetic Investigation of Communities by Reconstruction of Unobserved States (PICRUSt) program was used to predict the potential functions of each sample based on 16S rRNA high-throughput sequencing data. Then functional annotation and classification were carried out based on the Clusters of Orthologous Groups (COG) database [20].

### Accession numbers

The pyrosequencing datasets have been deposited into the NCBI Short Reads Archive Database (accession number: SRP467203).

## Results and discussion

### Abiotic control

Prior to biofiltration, an abiotic test was conducted to confirm the occurrence of biodegradation. The test used the blank BTF with the same packing materials and operating parameters, except for the fresh MSM supply and microorganism inoculation. The results indicated that the packing medium removed less than 1.53%, 1.78%, and 0.97% of the influent T, EA and H, respectively (S1 Fig). This suggested that biofiltration played a crucial role in removing the binary mixture of VOCs, rather than packing adsorption.

### Comparison of the effects of EA and H on the removal performances in BTF

During acclimation, laboratory-scale biofilters were usually fed with low concentrations of VOCs. In the present study, two parallel BTFs with an EBRT of 30 s were fed 300 mg m^-3^ of gas-phase T separately. After 15 days, a nearly stable RE value of 98.8% was achieved. Steady-state experiments were conducted to investigate the effect of EA on T removal in the BTF “A” after the acclimation period, as shown in Table 2.

**Fig 2:**
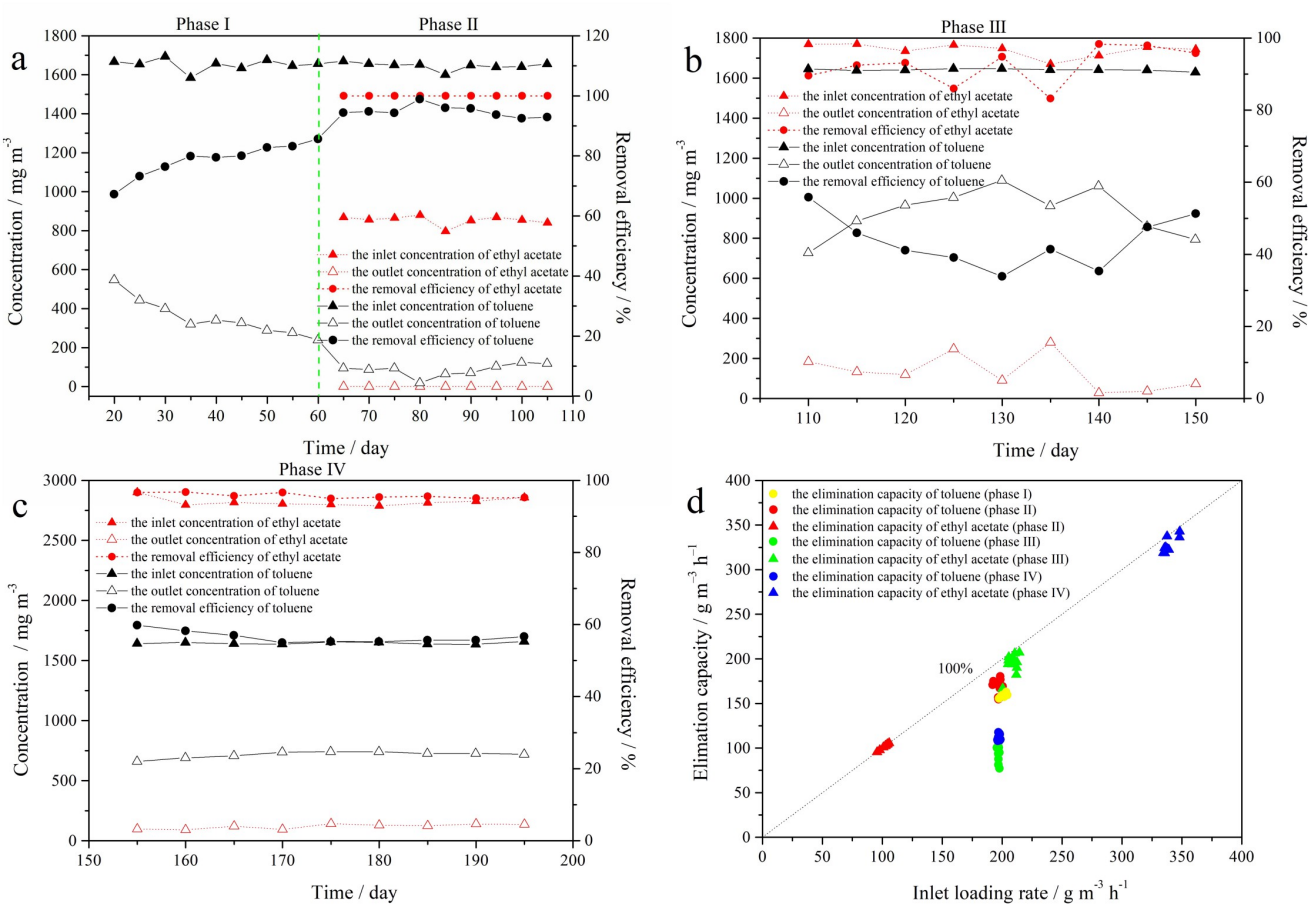
The REs (a-c) and EC/ILR (d) of a binary mixture of T and EA in the BTF “A” at different ratios. a: 1640 mg m^-3^ for T and 850 mg m^-3^ for EA, b: 1640 mg m^-3^ for T and 1750 mg m^-3^ for EA, c: 1640 mg m^-3^ for T and 2800 mg m^-3^ for EA, d: EC/ILR at different ratios. Each concentration was measured in triplicate.

During phase I (days 16–60), the T RE was 83.9 ± 1.7% when only 1640 ± 40 mg m^-3^ of T was fed to the BTF “A”. In phase II (days 61–105), EA (850 ± 55 mg m^-3^) was introduced to the BTF “A”, and the T concentration was maintained at 1640 ± 40 mg m^-3^. It was showed that the RE for T increased to 95.4 ± 2.2%, and 100% RE was achieved for EA at an EBRT of 30 s in Fig 2a. In phase III (days 106–150), the inlet concentration of EA increased from 850 ± 55 mg m^-3^ to 1750 ± 80 mg m^-3^, while the T concentration remained at 1640 ± 40 mg m^-3^. Compared to phase II, the RE for T decreased to 45.2 ± 7.7%, while most of the EA (93.0 ± 4.9%) was removed at an EBRT of 30 s (Fig 2b). In phase IV (days 151–195), 2800 ± 100 mg m^-3^ of EA and 1640 ± 40 mg m^-3^ of T were added to the BTF “A”. The REs for T and EA were 56.8 ± 1.6 and 95.9 ± 0.8%, respectively (Fig 2c). In phase II, the BTF “A” achieved a maximum EC of 180.3 g m^-3^ h^-1^ for T, followed by 162.7 g m^-3^ h^-1^, 117.7 g m^-3^ h^-1^, and 112.7 g m^-3^ h^-1^ in phase I, phase IV, and phase III, respectively. For EA, ECs of 105.6 g m^-3^ h^-1^, 207.1 g m^-3^ h^-1^ and 342.9 g m^-3^ h^-1^ were obtained during phase II, phase III, and phase IV, respectively (Fig 2d). The degradation of EA (EC vs IL) at 30 s EBRT was modelled using the equation y = 0.952x+1.53, with an R^2^ value of 0.9929, indicating a linear correlation. However, there was no linear correlation observed for T degradation.

The data suggested that low concentrations of EA can enhance T biodegradation, while medium and high concentrations have a negative impact on T biofiltration. This is consistent with the findings of Alvarez-Hornos et al. [21], who observed that EA inlet loads greater than 66 g m^−3^ h^−1^ inhibited T removal in the biofilter. The removal of EA in the BTF “A” was almost unaffected in the presence of T, which was consistent with the literature report by Zehraoui et al. [22].

**Fig 3:**
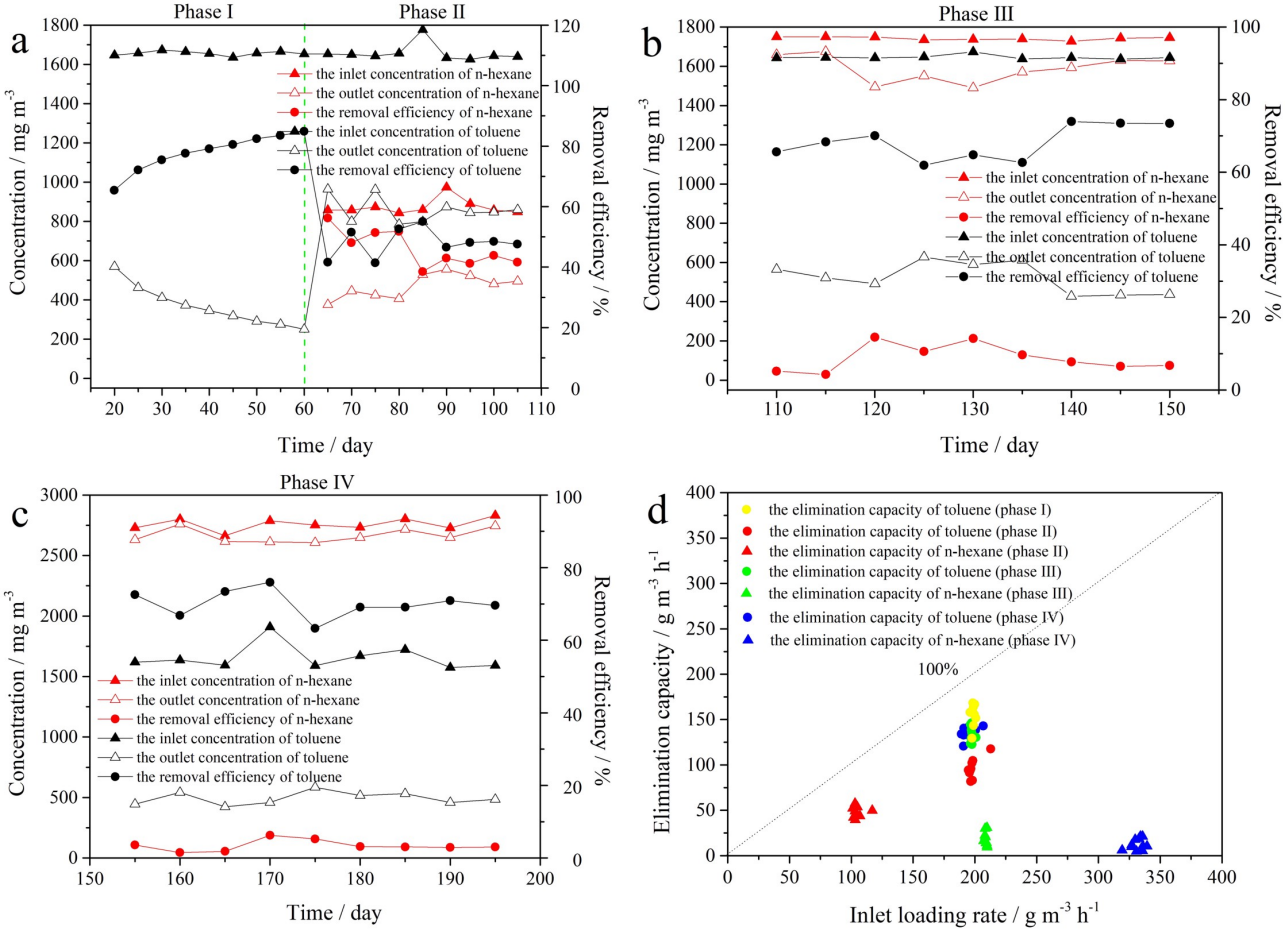
The REs (a-c) and EC/ILR (d) of a binary mixture of T and H in the BTF “B” at different ratios. a: 1640 mg m^-3^ for T and 850 mg m^-3^ for H, b: 1640 mg m^-3^ for T and 1750 mg m^-3^ for H, c: 1640 mg m^-3^ for T and 2800 mg m^-3^ for H, d: EC/ILR at different ratios. Each concentration was measured in triplicate.

Concurrently, BTF “B” was used to abate a binary mixture of T and H, as outlined in Table 2. During phase I (days 16–60), the T RE was 82.9 ± 2.9% when only 1640 ± 37 mg m^-3^ of T was fed to the BTF “B”. During phase II (days 61–105), 1640 ± 136 mg m^-3^ of T and 850 ± 123 mg m^-3^ of H were introduced into the BTF “B”. As shown in Fig 3a, the REs of T and H were 48.1 ± 6.9% and 46.2 ± 10.1%, respectively. During phase III (days 106–150), the inlet concentration of T was maintained at 1640 ± 34 mg m^-3^, while the concentration of H was gradually raised to 1750 ± 22 mg m^-3^ with the EBRT controlled at 30 s. Compared to phase II, the T RE increased to 68.3 ± 6.3%, while the H RE decreased to 8.8 ± 5.7% (Fig 3b). During phase IV (days 151–195), T-RE and H-RE were 70.1 ± 6.8% and 3.4 ± 2.9%, respectively, while the T concentration remained constant at 1640 ± 270 mg m^-3^ with an EBRT of 30 s. Meanwhile, the concentration of H increased to 2800 ± 136 mg m^-3^ (Fig 3c). In phase I, the BTF “B” received a maximum EC of 162.7 g m^-3^ h^-1^ for T, followed by 135.2 g m^-3^ h^-1^, 134.8 g m^-3^ h^-1^ and 95.8 g m^-3^ h^-1^ in phase IV, phase III and phase II, respectively, when T-IL was kept constant at 198.3±30.9 g m^-3^ h^-1^. The H-ECs were 48.3 g m^-3^ h^-1^, 15.2 g m^-3^ h^-1^ and 12.2 g m^-3^ h^-1^ in phase II, phase III, and phase IV, respectively, when the H-IL increased from 104.8 g m^-3^ h^-1^ to 331.7 g m^-3^ h^-1^ (Fig 3d). It showed that H had an inhibitory effect on the biodegradation of T in the BTF “B”. Iranmanesh et al. [23] demonstrated that T had a much greater inhibitory effect on the biodegradation of H than H had on T. Additionally, Hassan and Sorial [24] reported that the presence of benzene significantly affected the degradation of H, while the degradation of benzene was not impacted to the same extent as H. However, as the concentration of H increased, the negative impact on T removal gradually weakened. One reason is that the poor gas-liquid transfer of H, due to its high Henry’s constant, significantly limits the efficiency of mass transfer and biodegradation. In comparison, T is more readily utilized by microorganisms. Another reason is that T-degrading bacteria have a better reproduction with the extension of the running time.

### Comparison of carbon source distribution

The analytic results of the carbon balance are listed in Table 3. The carbon recoveries (*R*) in the biofiltration of mixtures of gas-phase T and EA were ranged from 97.1% to 101.2%,, indicating the accuracy of the test results. The majority of the effluent carbon was from CO_2_ production (59.2%, 81.1%, 53.9% and 60.4% in phase I, phase II, phase III, and phase IV, respectively), implying that the introduction of low concentration EA could improve the CO_2_ conversion rate of the binary mixture. As shown in Table 3, 20.3%, 12.1%, 8.4% and 11.9% of the influent carbon produced biofilm in phase I, phase II, phase III, and phase IV, respectively. This suggested that the carbon mass rate of the biomass was inhibited by the addition of EA. Furthermore, the data indicated that the dissolved VOCs and their derivatives in leachate were negligible in the BTF “A”.

**Table 3:** Carbon balance analysis of each phase.

**Phase**	***m*_*i*_ (mg C/min)**		***m*_*o*_ (mg C/min)**		**m_CO2_ (mg C/min)**	**m_biomass_ (mg C/min)**	**m_leachate_ (mg C/min)**	**R (%)**
**BTF “A”**	**T**	**EA**	**T**	**EA**				
**I**	24796	0	3992	0	14679	5034	372	97
**II**	24796	7671	1141	0	26331	3929	1457	101
**III**	24796	15794	13588	1106	21878	3410	325	99
**IV**	24796	25271	10712	1036	30240	5958	1570	99
**BTF “B”**	**T**	**H**	**T**	**H**				
**II**	24796	11782	12869	6339	11888	2634	2311	99
**III**	24796	24256	7860	22122	11969	4317	2034	99
**IV**	24796	38810	7414	37491	12403	4134	1311	99

For a binary mixture of T and H biofiltration, the Rs changed from 98.5% to 98.7%, while the MRs ranged from 19.5% to 32.5%. The rates of microbial assimilation fluctuated between 6.5% and 8.8%. The results showed that the addition of any concentration of H significantly inhibited T biodegradation.

### Microbial ecological analyses and correlation with the BTF performance

Samples of the packing beds were collected on days 60, 105, 150 and 195 representing phase I, phase II, phase III, and phase IV, respectively. The samples were preserved at −20°C and analyzed for the microbial communities using Illumina MiSeq high-throughput sequencing. Each sample yielded over 20,000 sequences, and the library size of each sample was normalized to the same bacterial sequencing depth (17,376 reads) by randomly removing the redundant reads for fair comparison.

**Fig 4:**
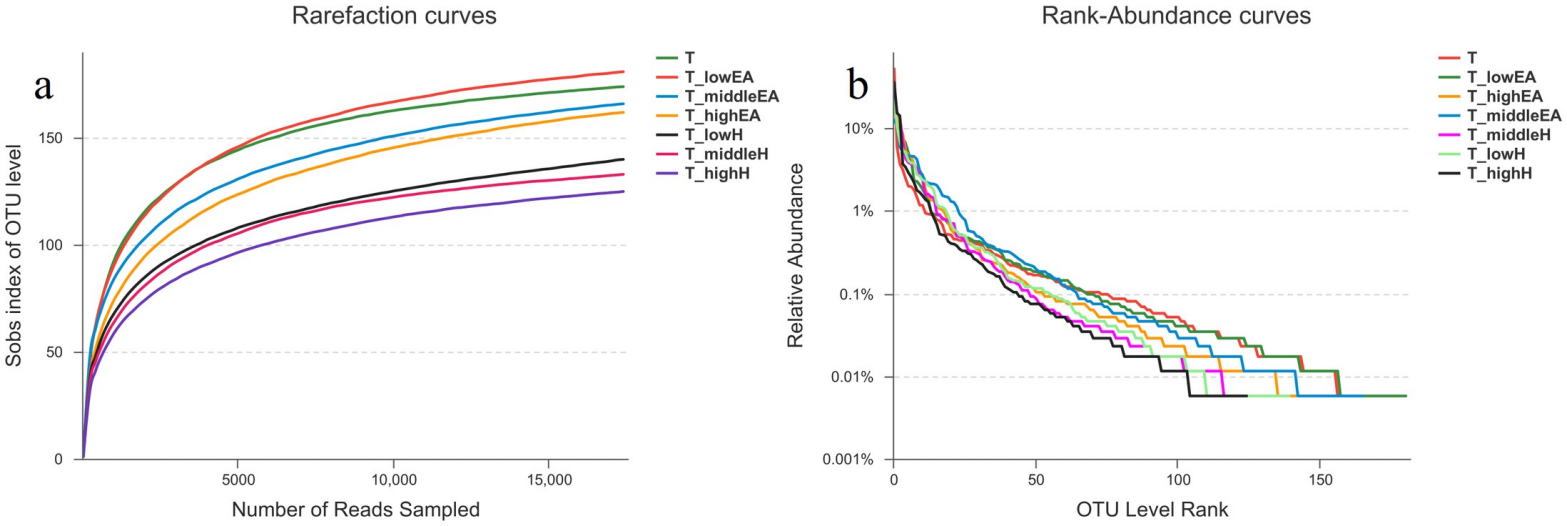
Rarefaction curves and rank-abundance curves based on OTU of the samples.

As depicted in Fig 4a, the Good’s coverage of these samples ranged from 99.83% to 99.90% at cutoff levels of 3%. This indicated that the bacterial community of these samples was well-represented by the generated sequences at this sequencing depth [19]. The species richness and evenness of phase II (toluene and low concentration of ethyl acetate, T_lowEA), which is similar to single T, were apparently higher than in other samples (Fig 4b). However, phase IV (toluene and high concentration of *n*-hexane, T_highH) had the lowest species richness and evenness.

**Fig 5:**
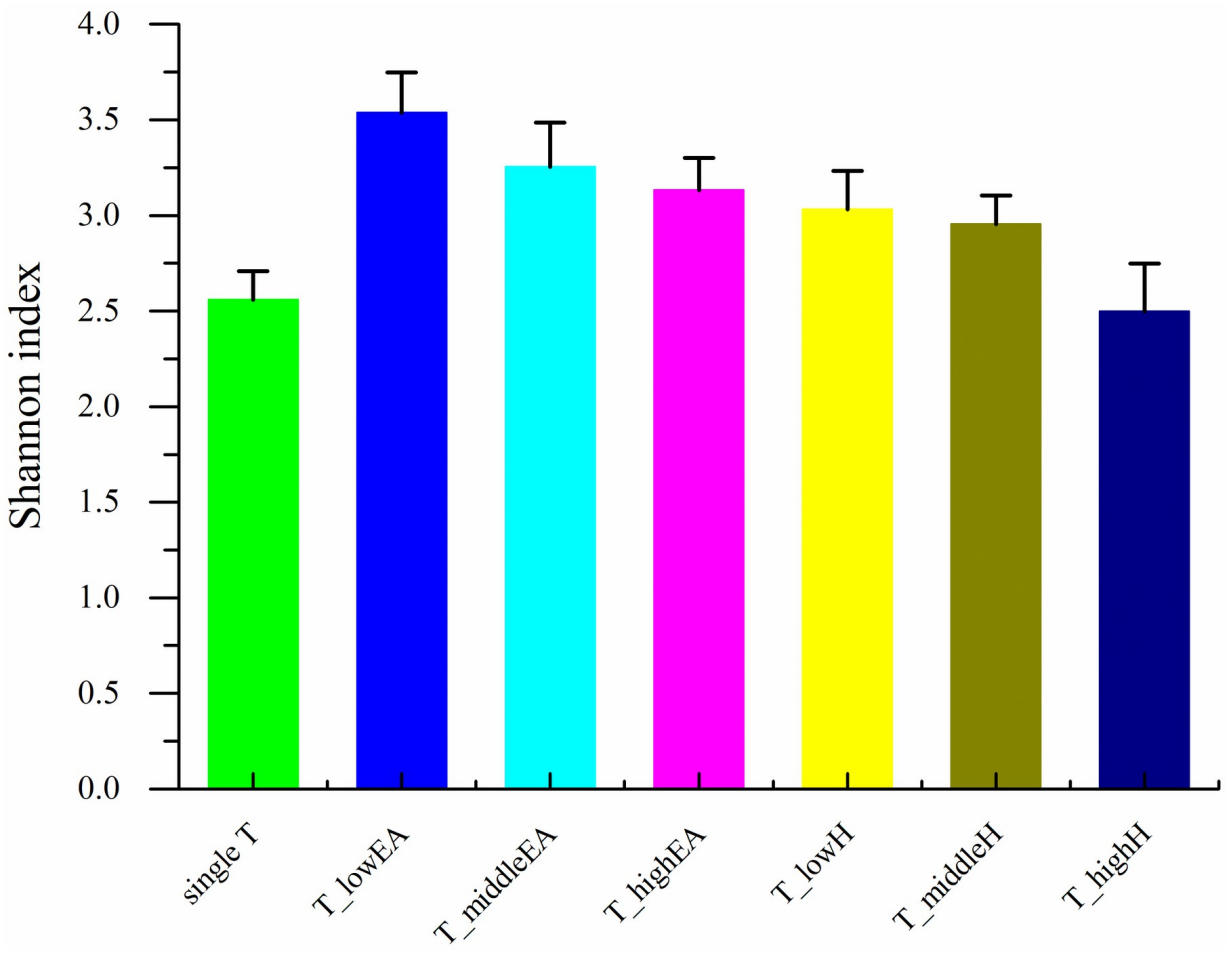
The Shannon index of the samples.

According to the Shannon index, T_lowEA (3.54) exhibited the highest microbial diversity among the samples, followed by phase III (toluene and middle concentration of ethyl acetate, T_middleEA) (3.25) and phase IV (toluene and high concentration of ethyl acetate, T_highEA) (3.13), which were higher than single T (2.56), as shown in Fig 5. This suggested that the presence of EA could enhance the species richness, evenness and microbial diversity of the BTF “A”, which was responsible for T removal. EA is a hydrophilic and easily biodegradable VOC that can provide readily accessible carbon sources for microbial growth. However, an oversupply of EA can alter the microbial community structure and inhibit the distribution and activity of T-degrading strains. For TBF “B”, the decrease in the Shannon index of the samples was observed as the concentration of H increased. The Shannon index of T_highH (2.50) was similar to that of single T (2.56), indicating that the impact on the species richness, evenness, and microbial diversity of the BTF “B” weakened with increasing H concentrations.

**Fig 6:**
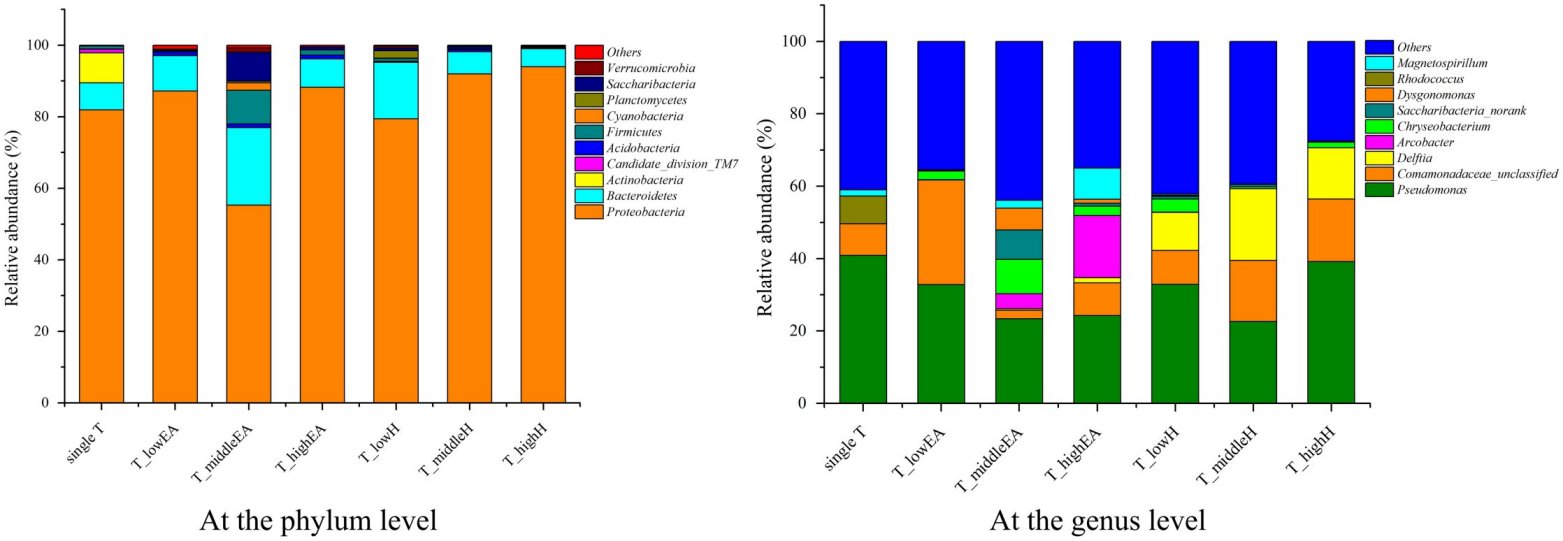
Microbial communities at the phylum and genus level.

The changes in the bacterial community structure at the phylum level in the samples were revealed by high-throughput sequencing technology, as illustrated in Fig 6a. The dominant flora were *Proteobacteria* and *Bacteroidetes*, which comprised 90% of the entire bacterial flora except in phase III (T_middleEA). Phyla *Proteobacteria* and *Bacteroidetes* were reported to be the dominant T-degrading and EA-degrading bacteria, respectively [25, 26]. During phase III of BTF “A”, the sum of the relative abundance of *Proteobacteria* and *Bacteroidetes* was only 76.9%. This led to a decrease in the removal performance, with a T-RE of 45.2 ± 7.7% and a T-EC of 112.7g m^-3^ h^-1^. Additionally, *Actinobacteria*, which was a major bacterial phylum in phase I, disappeared after the addition of EA. The abundance of *Acidobacteria* exceeded 1% in a binary mixture of T and EA during the removal of BTF “A”. The results indicated that the EA inhibited the growth of the phylum *Actinobacteria*, while promoting the proliferation of *Acidobacteria*. In the presence of H, the relative abundance of *Proteobacteria* and *Bacteroidetes* accounted for 95.2%, 98.2%, and 99.0% in phase II, phase III, and phase IV of BTF “B”, respectively. *Proteobacteria*, *Bacteroidetes* and *Actinobacteria* were the dominant bacterial phyla in the BTF for simultaneous removal of H and dichloromethane [27].

The microbial community composition at the genus level of the seven samples was shown in Fig 6b. During phase I for single T, phase II, phase III, and phase IV for T_EA of the BTF “A” and phase II, phase III, and phase IV for T_H of the BTF “B”, *Pseudomonas* (40.9%, 32.8%, 23.4%, 24.3%, 32.9%, 22.6%, and 39.2%, respectively) and *Comamonadaceae_unclassified* (8.7%, 28.9%, 2.3%, 9.0%, 9.3%, 16.9%, and 17.3%, respectively) were the most dominant co-existing bacteria. Therefore, the two bacteria played significant roles in the removal of T and EA / H. It is widely known that the genus *Pseudomonas* can degrade T under both aerobic and anaerobic conditions [28, 29] as well as EA [30, 31]. *Pseudomonadaceae* and *Comamonadaceae* have been reported as a typical T degradation genera [32]. For BTF “B”, another predominant genus was *Delftia*, which accounted for 10.5%, 19.8%, and 14.1% of the total microbial abundance of BTF for T_H mixture removal, respectively. *Delftia* has been reported to have the ability to degrade various aromatic compounds, such as naphenol, naphthalene, 2-methylnaphthalene, monochlorobenzene and T [33, 34]. Biodegradation of H by *Delftia* has not been reported to date. The results indicate that the presence of H did not have a significant effect on the T degradation activity of *Delftia*.

**Fig 7:**
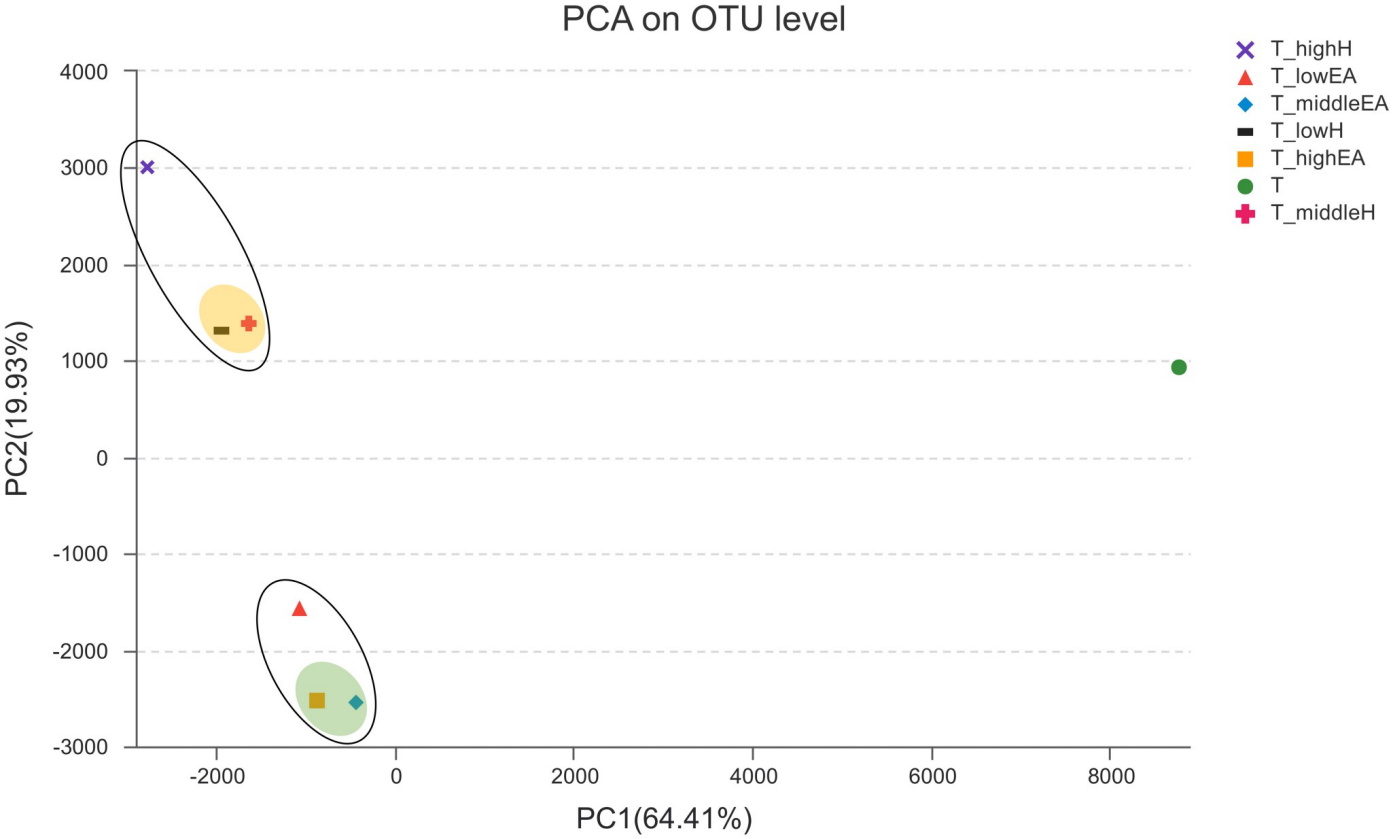
Principal coordinates analysis (PCoA) of the bacterial communities.

Principal coordinates analysis (PCoA) was used to illustrate the similarities and differences in the composition of the microbial community (Fig 7). In the PCoA plots, the T group is distributed on the right side, while the T_H groups are mostly located in the upper left corner, and the T_EA groups are located in the lower left corner. However, the T_highH group and T_lowEA group differed from the others. Therefore, the introduction of EA or H into the BTFs led to alterations in the diversity of the bacterial community. It indicated that different types and amounts of external VOCs may have varying effects on microbial communities of the BTF.

### Functional analysis in seven samples

**Fig 8:**
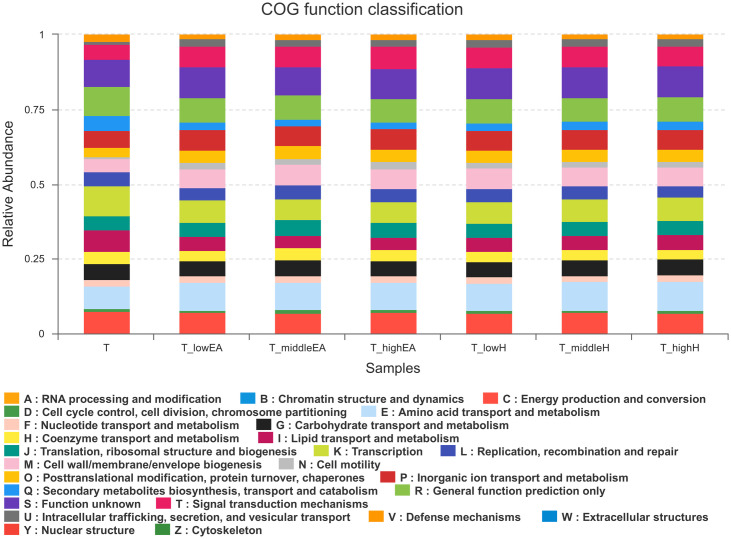
COG function classification of seven samples by PICRUSt analysis.

Using the methods mentioned in section 2.7, we obtained a microbial COG profile and correlated the microbial functional features with the important enzymes found in seven samples. The relative abundance of PICRUSt inferred function is illustrated in Fig 8.

Based on COG analysis, the microbial genes from seven samples were classified into 25 categories, as shown in Fig 8. In this classification, except for function unknown (S), the 10 most highly abundant functional genes of COG were as follows: amino acid transport and metabolism (E), which was followed by general function prediction only (R), transcription (K), signal transduction mechanisms (T), inorganic ion transport and metabolism (P), energy production and conversion (C), cell wall/membrane/envelope biogenesis (M), carbohydrate transport and metabolism (G), translation, ribosomal structure and biogenesis (J) and lipid transport and metabolism (I).

As illustrated in Fig 8, the expression of bacterial functional genes was similar across all seven samples, with only variations in the relative abundance of different genes. Following the addition of EA or H compared to single T, the relative abundance of E and P, which belong to metabolism, and T and M, which belong to cellular processes and signaling, increased. This suggests that biofilms are formed more easily and metabolism is more diverse in order to adapt to the pressure of mixed VOCs, which was consistent with the findings reported previously [35]. This is also supported by the Shannon index shown in Fig 5. However, the relative abundance of R, K and I, which belonged to the categories of Poorly Characterized, Information Storage and Processing, and Metabolism, respectively, tended to stabilize after a slight decline. This may be because that microorganisms develop defensive strategies to survive under the duress of mixed VOCs [36].

## Conclusions

Among the experimental conditions, BTF “A” achieved the highest removal performance for T when 850 ± 55 mg m^-3^ EA was introduced and T-IL was kept constant at 198.3 ± 30.9 g m^-3^ h^-1^, with a RE of 95.4 ± 2.2% and EC of 180.3 g m^-3^ h^-1^ at an EBRT of 30 s. The impact of H on the removal of T gradually weakened as the concentration of H increased from 850 ± 123 mg m^-3^ to 2800 ± 136 mg m^-3^ due to the hydrophobicity of H. High-throughput sequencing analyses suggested that *Pseudomonas* and *Comamonadaceae_unclassified* were the main contributors to the T degradation of T in BTF “A”, while *Pseudomonas*, *Comamonadaceae_unclassified* and *Delftia* are the main ones in BTF “B”. The introduction of low concentration EA resulted in an increase in the microbial diversity and the abundance of the genera *Pseudomonas* and *Comamonadaceae_unclassified*. However, the introduction of H has a significant negative impact on the mineralization rates of BTF “B”, as shown by the results of carbon balance analysis.

## Supporting information

S1 File Highlights. (DOCX)

S1 Raw data (XLSX)

S1 Fig An abiotic control. (TIF)

S2 Fig Graphical abstract. (TIF)
