# Supplementary material for: Biofiltration of toluene in the presence of ethyl acetate or n-hexane: Performance and microbial community
Source: PLoS One. 2024 May 7;19(5):e0302487. doi: 10.1371/journal.pone.0302487 (PMC11075902; doi:10.1371/journal.pone.0302487)
Supplement: S1 File — (DOCX) [file pone.0302487.s001.docx]

HIGHLIGHTS

- Biofiltration of toluene was conducted in the presence of ethyl acetate or *n*-hexane.
- The low concentration ethyl acetate improved BTF performance for toluene at an EBRT of 30 s.
- *n*-Hexane had an inhibition effect on toluene biodegradation.
- The BTF for the toluene removal has the highest species richness, evenness and microbial diversity in the presence of low concentration ethyl acetate.
- The high-throughput sequencing results showed that Genus *Pseudomonas and Comamonadaceae_unclassified* played a dominant role in a binary mixture of VOCs removal.
