# Supplementary figures and images for: Biofiltration of toluene in the presence of ethyl acetate or n-hexane: Performance and microbial community
Source: PLoS One. 2024 May 7;19(5):e0302487. doi: 10.1371/journal.pone.0302487 (PMC11075902; doi:10.1371/journal.pone.0302487)

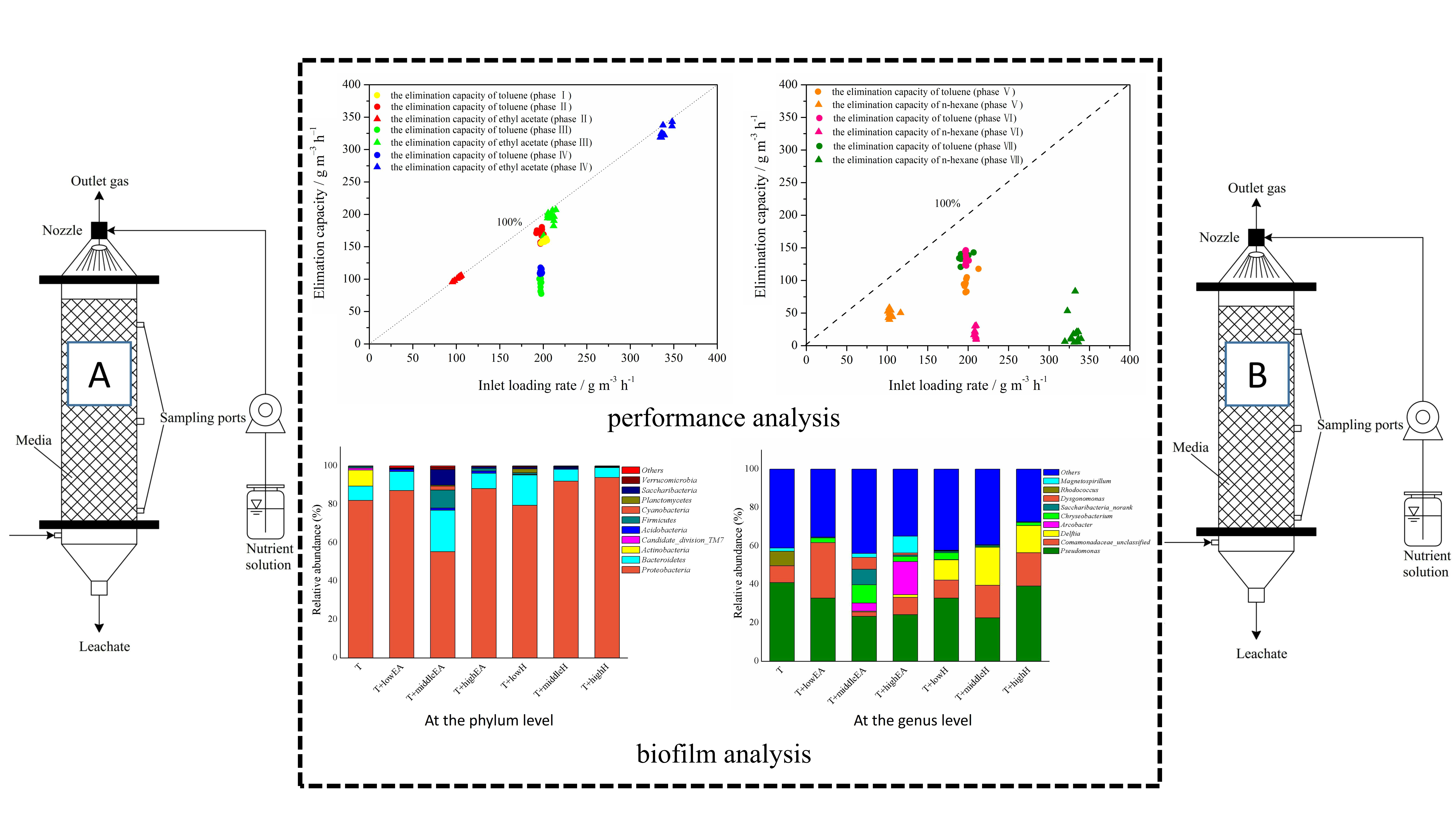

Supplement: S2 Fig — (TIF) [file pone.0302487.s004.tif]
